# Editorial: Qualitative research on therapist-client interaction in psychotherapy

**DOI:** 10.3389/fpsyg.2022.947374

**Published:** 2022-07-18

**Authors:** Yijin Wu

**Affiliations:** School of Translation Studies/Center for Medical Humanities in the Developing World, Qufu Normal University, Rizhao, China

**Keywords:** therapist-client interaction, psychotherapy, sequence, talk-in-interaction, qualitative analysis

In recent years, therapist-client interaction has drawn much attention to scholars who are interested in qualitative research (Peräkylä, 2019; Wu, 2019, 2021). Among the many choices of methods available to qualitative research on therapist-client interaction, Conversation Analysis (CA), Discourse Analysis (DA), and Critical Discourse Analysis (CDA) are distinct for their capacity to present the interactional details between therapist and client. In this collection, these qualitative methods have been applied to examine therapist-client interaction across different types of psychotherapy. Note that this collection involved a quantitative research, which was beyond the scope of this collection. That paper was recommended to us by the journal's editorial office.

Using the method of conversation analysis, Yao et al.'s research demonstrated how cognitive behavioral therapists ascribe agency positions to their clients by issuing formulations of what the clients have just said. Conversation analytic methodology analyzes in detail the concrete practices and actions that participants employ to achieve the outcomes at the turn-by-turn level. It does not view clients' agency, which has a key role in the therapeutic process, as a possession of an individual, but as a series of meaningful social actions that can be constructed and negotiated through talk-in-interaction. Under this framework, the authors deal with how the therapists ascribe agentic or non-agentic positions to their clients by issuing formulations of the clients' feelings-talk in the problem statement. In the interactional practice of formulating, the therapists addressed the clients' preceding problem-indicative turn by focusing on the emotion-relevant aspects of it in the formulation. Specifically, they identified two types of formulation: affirmative formulations and challenging formulations. Affirmative formulation was employed when the clients took a positive stance toward their experiences. On such occasions, the therapists formulated the positive side of the description and provided positive feedback on how well the clients managed the situation under discussion. In this case, the clients were formulated as active agents and thus were ascribed to an agentic position. Different from affirmative formulation, challenging formulation was used by the therapists when the clients took a negative emotional stance toward their own experiences. In challenging formulation, the therapists challenged clients' previous talk by transforming it into something that is apparently implausible, thus challenging the clients' dysfunctional thoughts and their non-agentic position.

Proceeding from a thorough review of relevant studies, Zeng et al. investigated agreement expression which is a commonly-used verbal strategy employed by the therapists in therapeutic conversation with children with ASD. Although this strategy was used with a high frequency in daily communication as well as therapeutic conversation, few studies have dealed with it in the context of psychotherapy. Based on the conversational data produced by five therapists and 10 Chinese Children with ASD, this study adopted the qualitative research method of CA to describe the three facets of the agreement expression, namely the strategies of expressing agreement, their sequential features as well as the intervention functions. The minute analysis shed light on some findings which may deepen our understanding of not only the expression itself but also the influence of the intervention method on therapeutic interaction in general. For instance, the study revealed that the therapists often expressed their agreement in the post-expansion positions, which demonstrated that the therapists intentionally used the strategy to perform functions like building supportive therapeutic relationship, serving as positive reinforcers, etc. The reasons behind the findings, as the authors proposed, were related to the intervention method of Naturalistic Intervention (NI), thus demonstrating the institutional influence of the intervention method on therapist talk.

Starting from the study on problem reformulation by Davis (1986), conversation analysis (CA) has been applied to the examination of the therapist-client interaction in psychotherapy. CA studies on psychotherapy not only focus on the machinery of the therapist-client interaction but the emotional and cognitive changes during the psychotherapeutic processes (Peräkylä, 2019). By adopting CA, Ma et al., were committed to the study of a relatively under-explored aspect of psychotherapeutic interaction in China, given that very few domestic studies in China have been conducted on the interaction between therapists and adolescents with depression.

Scheidt et al., conducted a qualitative review of the importance of movement synchronization for adult psychotherapy and proposed approaches to measuring movement synchronization in psychotherapy. It has been indicated that the meaning of the synchrony research in psychotherapy could help people understand the “procedural dynamics of the therapeutic relationship.” In order to understand the different roles of movement synchrony in solving communicative tasks, it is necessary to analyze the relationship between specific instances of synchrony and its semantic context and content. Thus, a set of criteria for classifying various synchrony phenomena have been proposed, which could be beneficial to measuring movement synchronization in psychotherapy.

Akimoto et al. investigated the neural mechanism of therapeutic alliances in Sandplay therapy. Specifically, the authors adopted an experimental method to examine the collected data. It has been identified that correlations exist between lateral prefrontal cortex and frontopolar regions. Galasiński et al., used the method of Critical Discourse Analysis (CDA) to analyze how clients describe the meaningful values brought to them by the journey to psychotherapy sessions, and how the absence of the journey to psychotherapy were addressed. The authors concentrated on the discourses therapists and their clients used when they interact with each other. Semi-structured interviews have been used to examine: (1) experience of online psychotherapeutic talk; (2) circumstances of the first online psychotherapy; (3) consequences of the change of communication channel. It has been suggested that clients tend to portray themselves as agents when they narrate their experience on their journey to psychotherapy sessions.

This collection could help readers understand how psychotherapy is interactionally achieved by therapist-client interaction. Also, the research findings above could shed insights on how a therapeutic alliance was sequentially and interactionally achieved in actual psychotherapy.

## Author contributions

The author confirms being the sole contributor of this work and has approved it for publication.

## Funding

This research was supported by Shandong Social Science Planning Fund Program [Grant ID: 21CYYJ08].

## Conflict of interest

The author declares that the research was conducted in the absence of any commercial or financial relationships that could be construed as a potential conflict of interest.

## Publisher's note

All claims expressed in this article are solely those of the authors and do not necessarily represent those of their affiliated organizations, or those of the publisher, the editors and the reviewers. Any product that may be evaluated in this article, or claim that may be made by its manufacturer, is not guaranteed or endorsed by the publisher.
